# Academic and Demographic Characteristics of Orthopedic Adult Reconstruction Division Chiefs

**DOI:** 10.7759/cureus.62612

**Published:** 2024-06-18

**Authors:** Anshu Punreddy, Paul Guirguis, Ankit Punreddy, Mark Youssef, Adwin Denasty, Mina Botros

**Affiliations:** 1 Medicine, George Washington School of Medicine and Health Sciences, Washington, DC, USA; 2 Orthopedics, University of Rochester School of Medicine and Dentistry, Rochester, USA; 3 Plastic Surgery, University of Rochester School of Medicine and Dentistry, Rochester, USA; 4 Orthopedics, A.T. Still University, Mesa, USA; 5 Orthopedics, University of Rochester Medical Center, Rochester, USA; 6 Orthopedic Surgery, University of Rochester Medical Center, Rochester, USA

**Keywords:** division chiefs, demographics, orthopedics, arthroplasty, adult reconstruction

## Abstract

Introduction: Division chiefs play crucial leadership, administrative, and instructive roles within orthopedic subspecialties. The purpose of this study is to investigate the demographic and academic characteristics of division chiefs of adult reconstruction at fellowship institutions in the United States.

Methods: Adult reconstruction fellowship programs were identified using the American Association of Hip and Knee Surgeons database. Characteristic information about sex, race, academic rank, additional degrees, fellowship institution, and year of completion were collected. Hirsch indices (h-indices) of the division chiefs were collected from the Scopus database.

Results: Of the 120 adult reconstruction fellowship programs identified, 39 had a designated division chief of adult reconstruction. All of the division chiefs were male (n=39). Race breakdown was as follows: 74.4% were White (n=29), 12.8% were Asian (n=5), 7.7% were of mixed ethnicity (n=3), 2.6% were Latinx (n=1), and 2.6% were African American (n=1). The majority (53.8%; n=21) of division chiefs also held the academic rank of professor. The mean time since completion of fellowship was 21.7 ± 8.2 years and the mean h-index of the division chiefs was 24.9 ± 16.2. The fellowship programs that trained the most division chiefs were Massachusetts General Hospital (n=9) and the Hospital for Special Surgery (n=6).

Discussion: Division chiefs of adult reconstruction are integral leaders within their orthopedic subspecialty. An analysis of demographic and educational characteristics revealed a lack of diversity among adult reconstruction division chiefs in the United States. Deliberate efforts to increase the diversity of adult reconstruction leadership must be made to address these disparities.

## Introduction

Adult reconstruction is an important subspecialty of orthopedics that aims to treat musculoskeletal diseases, degenerative conditions, or traumatic injuries of the hip or knee. Total hip and knee arthroplasty surgeries are two of the most common procedures performed in the United States, with an estimated 7 million Americans living with a total hip or knee replacement and over 1 million total hip and knee replacements performed each year [1,2].

As a subspecialty of orthopedics, the adult reconstruction division offers several leadership positions, including division chiefs and fellowship directors. The adult reconstruction division is typically headed by the division chief, who works alongside the leadership of the department of orthopedic surgery. In addition to their clinical responsibilities, these division chiefs are also often involved in creating institutional and national guidelines alongside other administrative duties. They may also serve dually as the division chief and the fellowship director. As such, a study of the demographics and academic characteristics of adult reconstruction division chiefs is important, as these leaders hold immense responsibility and influence regarding institutional policies and goals. To date, there are minimal publications about adult reconstruction division chiefs; however, there have been studies on the division chiefs of other orthopedic subspecialties, including sports medicine [3], spine surgery [4], and pediatric orthopedics [5]. Other medical fields, such as plastic and reconstructive surgery ​​[6], cardiovascular disease [7], and pulmonary and critical care medicine [8], have also studied the demographics and characteristics of their division chiefs. These studies found that the division chiefs were primarily internal hires and academically ranked males [6,7].

The objectives of this study were to (1) record the academic and demographic characteristics of adult reconstruction division chiefs, (2) identify any patterns in this characteristic information, and (3) compare the results to previous studies on division chiefs of other specialties.

## Materials and methods

Study design

This is a cross-sectional analysis of division chiefs of adult reconstruction at institutions with adult reconstruction fellowship programs. This study was exempt from institutional review board approval as it only involved publicly derived information without the use of human subjects, deeming it Health Insurance Portability and Accountability Act compliant.

The American Association of Hip and Knee Surgeons (AAHKS) database was used to identify institutions with adult reconstructive hip and knee fellowship training programs. In May 2024, the fellowship programs with division chiefs for adult reconstruction departments were identified using each institution’s public institutional websites. Faculty profiles on institutional websites were then utilized to obtain division chief demographics and educational characteristics.

Using these websites and faculty profiles, the sex, race, academic rank, additional degrees obtained, fellowship institution, and years since completion of fellowship were determined for each division. Author names were also searched on Google, and other websites were utilized to corroborate or gather missing information. The Hirsch index (h-index), a metric that measures the productivity and impact of an author’s publications, was also determined for each division chief as of May 2024 using the Scopus database. An index of h indicates that h of the author’s publications have been cited ≥h times [9]. Discrete variables were reported as counts and proportions. Continuous variables were reported as means with standard deviations.

Inclusion and exclusion criteria

Faculty met the inclusion criteria for the study if they were listed as the division chief of adult reconstruction, division chief of arthroplasty, or division chief of joint replacement on their institution’s academic website. Individuals were excluded from the study if they did not have these specific professional titles.

Statistical analysis

Microsoft Excel (Microsoft Corporation, WA, USA) was used to collect, analyze, and visualize the data. Discrete variables were reported as counts and proportions. Continuous variables were reported as means with standard deviations.

## Results

One hundred twenty adult reconstruction fellowship programs were identified, 39 of which had division chiefs of adult reconstruction listed on their institutional websites. All division chiefs were male (n=39; 100%). Race and ethnicity were as follows: 74.4% White (n=29), 12.8% Asian (n=5), 7.7% mixed ethnicity (n=3), 2.6% African American (n=1), and 2.6% Latinx (n=1).

**Figure 1:**
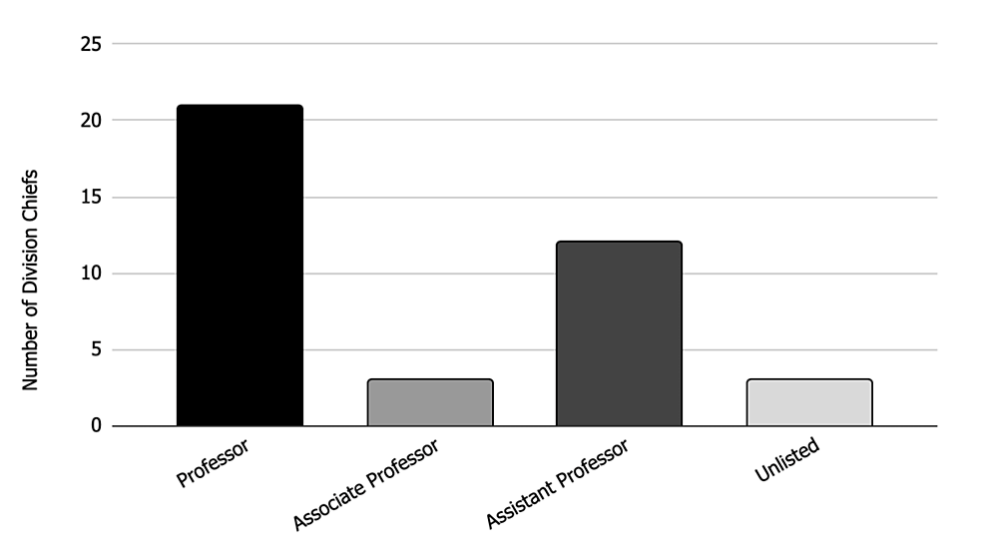
Academic rank of division chiefs Bar chart showing the academic ranks of the adult reconstruction division chiefs at their respective institutions. "Unlisted" indicates that the academic rank of the division chief was not provided on any academic websites or that the division chief held no academic rank.

Twenty-one (53.8%) of the division chiefs held the academic rank of professor, three (7.7%) held the rank of associate professor, 12 (30.8%) held an assistant professor rank, and three (7.7%) did not have an academic rank listed (Figure 1). Of the 39 division chiefs, 16 (41.0%) were also the directors of the adult reconstruction fellowships at their current institution.

**Figure 2:**
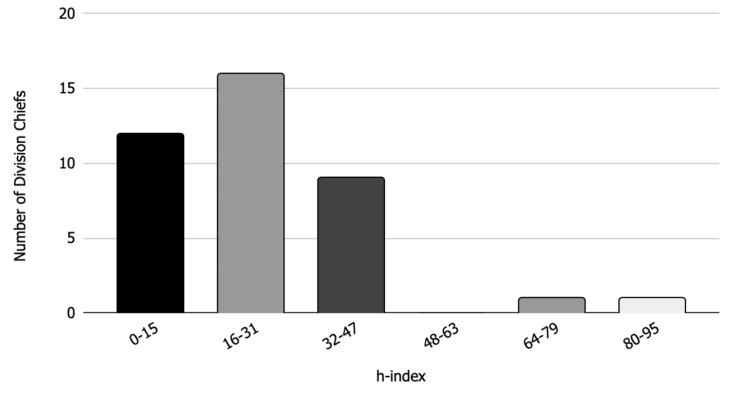
h-indices Bar chart showing h-indices of adult reconstruction division chiefs.

Only seven (17.9%) of the division chiefs had additional degrees, of which four (10.3%) had master's degrees, two (5.1%) had their master’s in business administration, and one (2.6%) had a doctorate of philosophy. The mean number of years since adult reconstruction fellowship completion was 21.7 ± 8.2 years. The mean h-index was 24.9 ± 16.2 (Figure 2).

**Figure 3:**
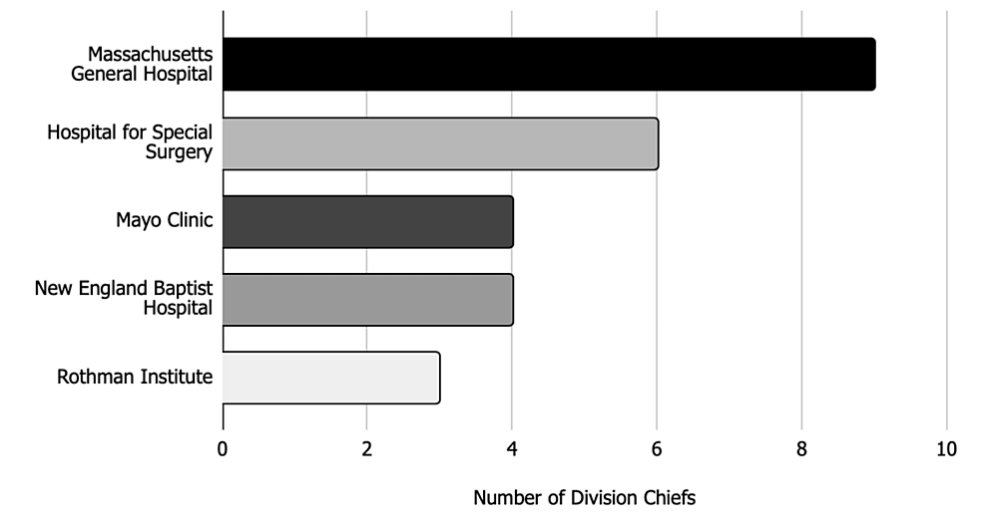
Most common fellowships attended by division chiefs Bar chart showing fellowship institutions that trained three or more adult reconstruction division chiefs.

The adult reconstruction fellowships that trained three or more of the current adult reconstruction division chiefs were identified. These included Massachusetts General Hospital (n=9), Hospital for Special Surgery (n=6), Mayo Clinic (n=4), New England Baptist Hospital (n=4), and Rothman Institute (n=3) (Figure 3).

## Discussion

Division chiefs of adult reconstruction are important leaders of the orthopedic subspecialties tasked with heading the division, policy-making, and performing other administrative roles. There is currently a lack of available publications regarding the demographics and professional backgrounds of adult reconstruction division chiefs. This is the first study that aims to evaluate the academic and demographic characteristics of adult reconstruction division chiefs.

Adult reconstruction division chiefs had completed their fellowship training an average of 21.7 years prior, with the majority holding the academic title of professor. Data on the time between completion of fellowship training and appointment as division chief were not readily available; however, this information may be beneficial for future studies. This result highlights the professional and clinical experience required to perform the leadership and teaching duties of a division chief. Furthermore, 41% of the division chiefs served as directors of their institution’s adult reconstruction fellowship program.

**Figure 4:**
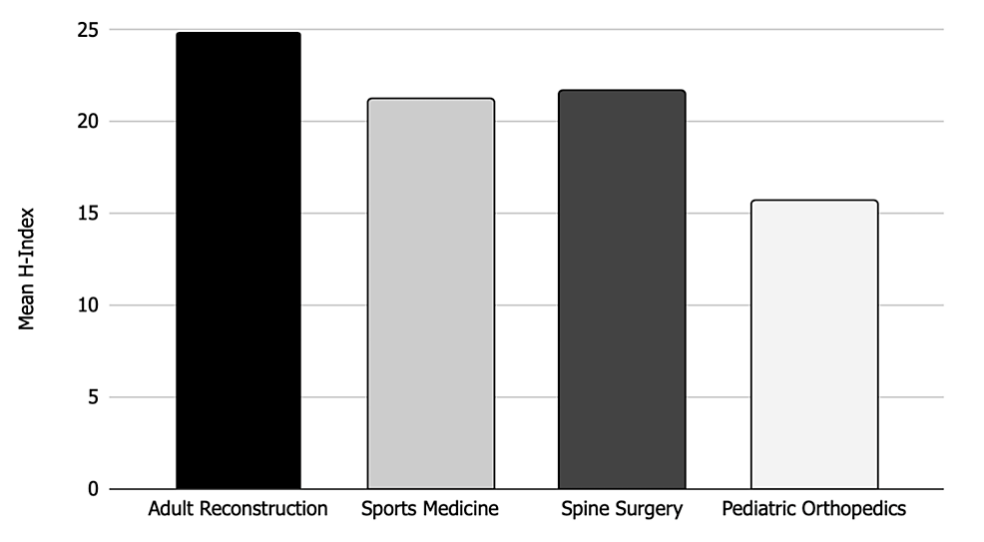
Mean h-indices for orthopedic subspecialties Bar chart showing mean h-indices for division chiefs of various orthopedic subspecialties.

In regards to academic productivity, the mean h-index for the adult reconstruction division chiefs was 24.9, which was similar but slightly greater than the mean h-index for division chiefs of other orthopedic subspecialties reported in prior analyses. Previous studies reported that sports medicine division chiefs had a mean h-index of 21.2 [3], spine surgery division chiefs had a mean h-index of 22.1 [4], and pediatric orthopedic division chiefs had a mean h-index of 15.7 (Figure 4) [5]. Additionally, one prior study on the productivity of orthopedic surgery faculty in the United States found that the mean h-index for orthopedic surgeons was 5 [10]. It is not surprising that the mean h-index for the division chiefs is decidedly higher than the mean for orthopedic surgeons, given the additional experience and knowledge required for a leadership position.

Notably, the demographic results of this study illustrated opportunities for improvement in diversity among adult reconstruction division chiefs. One hundred percent of the division chiefs were male, and only 25% were non-White. A previous study on the demographics of adult reconstruction fellowship directors found that 100% of the fellowship directors were male and 80.6% were White [11]. Previous studies on sports medicine [3], spine surgery [4], and pediatric orthopedic [5] leadership positions also found the majority of division chiefs to be male (85.7-99%) and White (72.5-86%). This disparity is also present among division chiefs in other specialties, including plastic and reconstructive surgery [6], cardiovascular disease [7], and pulmonary and critical care medicine [8].

The results of the present study highlight gender and racial disparities within leadership positions among orthopedic subspecialties. Prior studies regarding the demographics of orthopedic surgeons have shown that these disparities persist and that diversity has been slower to progress within the orthopedic field compared to other fields [12]. Increasing diversity within medicine is crucial, as previous studies have shown that underrepresented minority physicians can help improve access to healthcare and healthcare satisfaction for minority patients [12]. These physicians can also serve as role models for younger, aspiring physicians to further diversify the medical field. Mentorship and early exposure have proven vital for diversifying interest in orthopedic surgery [12].

This study had several limitations. First, the majority of the adult reconstruction fellowship programs from the AAHKS database did not have division chiefs of adult reconstruction listed on institutional websites. The small sample size may hinder comparisons with the fellowship directors and division chiefs of other specialties. Second, the demographic and academic characteristics collected were limited to data reported on institutional pages, which may not be consistently updated. Finally, institution identification was based on the AAHKS fellowship program list, which excludes institutions without reconstruction training programs. Some institutions may utilize other titles for division chief positions that were not included in this analysis.

## Conclusions

An analysis of adult reconstruction fellowship institutions revealed that the majority of division chiefs held high academic ranks and had over two decades of post-fellowship experience. In addition, adult reconstruction division chiefs were well-published and cited more often compared to other orthopedic subspecialties. Lastly, women and non-White faculty were underrepresented as division chiefs, illustrating a potential opportunity for enhancing diversity within the field.
